# Antimicrobial and optical properties of a new biogenic silica-coated silver nanoparticles incorporated into experimental resin 

**DOI:** 10.4317/jced.61004

**Published:** 2024-02-01

**Authors:** Marina-Mariante Viana, Thaís-Rodrigues Souza, Bruno Bueno-Silva, Flávia Gonçalves, Roberto-Ruggiero Braga, Fábio-Dupart Nascimento, Rodrigo-Mendes Pereira, Bruno-Lemos Batista, Amedea-Barozzi Seabra, Marcela-Charantola Rodrigues

**Affiliations:** 1 Doctoral student, Cruzeiro do Sul University, São Paulo, Brazil; 2 Post-graduate student, Cruzeiro do Sul University, São Paulo, Brazil; 3 Assistant Professor, Guarulhos University, Guarulhos, Brazil; 4 Assistant Professor, Santo Amaro University, São Paulo, Brazil; 5 Full Professor, Department of Biomaterials and Oral Biology, University of São Paulo, São Paulo, Brazil; 6 Assistant Professor Department of Biochemistry/Molecular Biology Division, Federal University of São Paulo, Brazil; 7 Postdoctoral student, Center for Natural and Human Sciences (CCNH), Federal University of ABC, Santo André, Brazil; 8 Associate Professor, Center for Natural and Human Sciences (CCNH), Federal University of ABC, Santo André, Brazil; 9 Assistant Professor, Municipal University of São Caetano do Sul (USCS), São Caetano do Sul, Brazil

## Abstract

**Background:**

Evaluate the effects of incorporating silica-coated silver nanoparticles (Ag@SiO2 NPs) into odontological clinic resin materials.

**Material and Methods:**

Silver nanoparticles coated with silicon dioxide were added to the experimental resin matrix at 1, 3, and 5wt%. Degree of conversion (DC), optical properties (total transmittance and color change), and microstructural analysis were evaluated. Materials were tested for silver ion release, cytotoxicity in dental pulp fibroblasts, Streptococcus mutans biofilm growth by Colony-Forming Unit (CFU) and confocal laser scanning microscopy (CLSM).

**Results:**

Groups had a similar DC, despite significant differences observed in transmittance and color change analysis for all groups with NPs. Silver ion release values were below the detection limit after 72h for all groups, and NPs incorporation did not show a statistical difference from the control on pulp fibroblasts assay. After 72h, the CFU count was significantly reduced by 74% from 3wt% of Ag@SiO2NPs. CLSM evaluation on *S. mutans* colonies showed a dose-dependent decrease in the emitted fluorescence.

**Conclusions:**

The application of Ag@SiO2 NPs in a resinous matrix, demonstrates a significant reduction of *S. mutans* CFU in oral biofilm, at concentrations from 3wt%, without an increase in cytotoxicity. The reduced transmittance values did not affect the DC, although a significant color change was perceived in all concentrations.

** Key words:**Nanoparticles, Silver Compounds, Composite Dental Resin, Anti-Bacterial Agent, Optical Imaging.

## Introduction

Several compounds have been evaluated as antimicrobial agents to be incorporated into composite resins, such as particles derived from quaternary ammonium (1,2), antimicrobial monomers (3-5), bioglass (6,7), chlorhexidine (8), zinc oxide (9,10), titanium dioxide (11), chitosan (12,13) and silver nanoparticles (3,14). These antimicrobial compounds are expected to have specific toxicity (14) with an effective spectrum of action and lasting effect (3,15). Ideally, their incorporation in the restorative materials should not affect their mechanical (7,10,15) and/or optical(10) properties.

Silver nanoparticles (AgNPs) have shown favorable results in controlling and preventing microbial adhesion to dental materials (16,17). The antimicrobial effect of these particles had been observed in multi-species biofilm models (18,19), and its effectiveness against *Streptococcus mutans* (9,20-23) and *Lactobacillus* (9,21), is well established. However, there is still no consensus on the antimicrobial mechanism of silver nanoparticles. More recently, it has been described as a combination of mechanisms involving ion release (Ag+) with reactive oxygen species production, and accumulation and interaction of nanoparticles in the cell membrane that could affect its function and permeability (24).

The incorporation of antimicrobial agents should not compromise the material’s mechanical properties. However, there is a concern about the risk of incomplete polymerization of resin-based materials containing AgNPs.

Regarding optical properties of the material, it is predicted that the addition of silver nanoparticles into resin materials, will cause significant color changes (25,26). In fact, significant reductions in light transmission values (from 593 to 48mW/cm2) were observed after the incorporation of 2000 ppm of Ag in the polymer (25). Significant color changes (∆E), up to 32, were also measured upon incorporation of 20 or 30% by mass of calcium phosphate hybrid particles containing silver (1.2% by mass of Ag). The observed values of ∆E were up to 32, using a commercial composite resin of color A3 as parameter (26).

The coating of AgNPs with silicon dioxide (Ag@SiO2 NPs) has been described in the literature as a possibility to obtain a silver compound with a more compatible color for application in dental materials. In a preliminary study, the synthesis, characterization, and initial biological evaluation of coated nanoparticles was described, and a potent antimicrobial effect against the pathogen *Streptococcus mutans* in oral biofilm was observed at low concentrations of Ag@SiO2 NPS (27). In this follow-up study, the effects of incorporating Ag@SiO2 NPs into resin materials on the antimicrobial, ion release and cytotoxic effects of the materials were tested. In addition, the degree of conversion and optical properties were evaluated. The working hypothesis was that the coating of AgNPs with SiO2 NPs e would allow the addition of antimicrobial activity to resin materials without compromising their optical properties.

## Material and Methods

-Manipulation of resin materials 

An experimental resin matrix was prepared with BisGMA (2,2bis[4-(2-hidroxi-3-metacriloxipropoxi)fenil]-propane, Sigma-Aldrich Inc.) and TEGDMA (2-methyl 2- propenoic acid, Sigma-Aldrich Inc.) at 1:1 (mol), with photoiniciators dimethylaminoethyl acrylate (DMAEMA, Sigma-Aldrich Inc.) and camphorquinone (Sigma-Aldrich Inc.) at 0.5 wt%.

**Figure 1:**
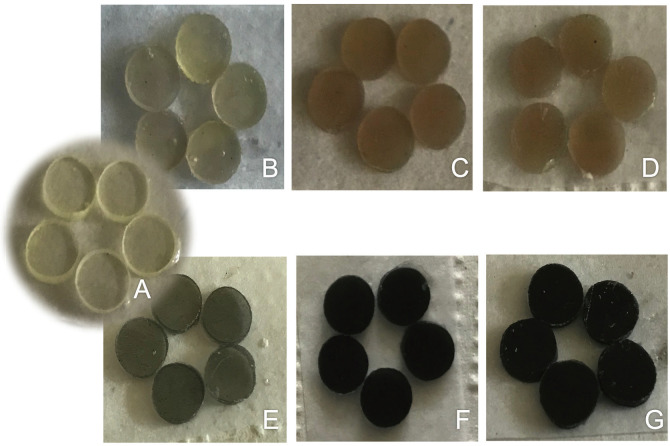
Pilot samples of the experimental resin development groups. A, Control material without particles. B, Experimental material with 1wt% of Ag@SiO2 NPs. C, Experimental material with 3wt% of Ag@SiO2 NPs. D, Experimental material with 5wt% of Ag@SiO2 NPs. E, Experimental material with 1wt% of GT-AgNPs. F, Experimental material with 3wt% of GT-AgNPs. G, Experimental material with 5wt% of GT-AgNPs.

Initially, samples were prepared with resin containing 1, 3 or 5 wt% of green tea silver nanoparticles (GT-AgNPs) or silver nanoparticles coated with silicon dioxide (Ag@SiO2 NPs) previously synthesized and characterized (27). Nonetheless, the addition of GT-AgNPs into resin material resulted in extremely dark materials, as shown in Figure 1. Therefore, only nanoparticles coated with silicon dioxide were used. A group without nanoparticles was kept as control. All materials were mechanically mixed under vacuum (Speedmixer DAC 150.1 FVZeK, FlackTek Inc.) and kept under refrigeration until 2 h before the use.

-Degree of conversion 

**Figure 2:**
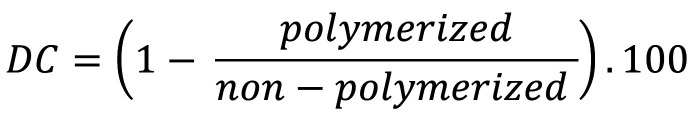
Formula.

Degree of conversion was determined by Fourier-transformed Infrared Spectroscopy (FT-IR). Spectra from samples (n=3) 0.9-mm thick and 5 mm diameter were obtained (Vertex 70, Bruker Optik GmbH) before polymerization at wavelengths between 4000 cm-1 and 9840 cm-1, and a 6 cm-1 resolution. Photopolymerization was performed using a LED unit (Radii-Cal, SDI) with an energy density of 48 J/cm2 (1200 mW/cm2 x 40 s), and the samples were kept at 37°C under relative humidity of 100% for 24 h, when a new spectrum of the material was collected. The area under the absorption band of the vinyl bond at 6165 cm-1 was determined using Opus v.6 software (Bruker Optics) and the degree of conversion was calculated by the formula, (Fig. 2):

-Optical Properties

Total transmittance and color change of the experimental resin disk samples (n=12; prepared as previously described) were evaluated through spectrophotometry (CM-3700d, Konica Minolta). For the analyses, the specimens were positioned perpendicular to the visible light beam wavelength range (360 to 740 nm) radiation emission generated by a xenon arc lamp. Each disk was analyzed in triplicate, and the average values were used for calculation. The transmittance values in 470 nm were generated by the OnColorTM QC Lite (T & M Instruments) software, obtaining the average values for each wavelength.

**Figure 3:**
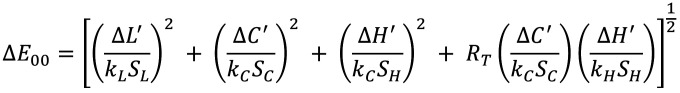
Formula.

Color change (ΔE00) among the experimental resins containing different percentages of Ag@SiO2 NPs was calculated based on the values of CIEDE2000 color system, according to the following equation (28), (Fig. 3):

where ∆L´, ∆C´ and ∆H´ are the differences in lightness, chroma, and hue respectively; SL, SC and SH are weighting functions used to adjust the total color difference; kL, kC and kH are corrections terms for experimental conditions and RT is the rotation function that accounts for the interaction between chroma and hue differences in the blue region. ∆E00<1.8 is an acceptable color difference threshold for the CIEDE2000 method (29).

-Microstructural Analysis

Resin disks surfaces were analyzed in scanning electron microscopy (Quanta 650 FEG, Thermo Fisher Scientific) in back-scattered electron mode. A minimum of 10 particles from each resin group was evaluated (size and shape) using Image J software (National Institutes of Health).

-Silver Ion Release

For the evaluation of silver ion (Ag+) release, culture medium samples from the biofilm growth assay that were in contact with the resin specimens for 72 h were collected after 24, 48, and 72 h of the experiment. The total silver contents in these samples were determined using an inductively coupled plasma mass spectrometer (ICP-MS 7900, Agilent) 

-Fibroblasts isolament

The human fibroblasts from dental pulp isolament and use was approved by the Research Ethic Committee (CAAE n° 25305019.3.0000.8084 and nº 25305019.3.3001.5597). Primary culture of fibroblasts was obtained from human dental pulp fragments, through the explant technique. Human permanent teeth with surgical indications were removed under local anesthesia after free and informed consent of the patient and transported to the lab in DMEM with 10% penicillin/erythromycin (10000U mLmL-1). The tooth was abundantly washed with PBS (pH 7,2) and cut with a diamond disc, to separate the crown from root. The dental pulp was removed using an endodontic file under PBS refrigeration. The tissue was cut into small fragments with a blade no 15, immersed in trypsin, and incubated at 37ºC with 5% CO2 for 5 min. The trypsin was inactivated with DMEM with 10% Dulbecco’s Modified Eagle Medium (DMEM – Vitrocell) containing 10% fetal bovine serum (FBS - Vitrocell) and 1% penicillin/erythromycin (10000U mLmL-1), and the explants were maintained in cultured. The fibroblasts cells that migrated from the explant to the culture dishes were removed with trypsin, transferred to culture bottles where they were cultured, and expanded. The cultures were maintained in semi-confluence until their use or freezing.

-Cytotoxicity Assay

This test ran according to the ISO 10993-5. Sample disks (n = 3; prepared as described in the previous assay) from each group were immersed, for 24 h, in Dulbecco’s Modified Eagle Medium (DMEM – Vitrocell) containing 10% fetal bovine serum (FBS - Vitrocell) and 1% penicillin/erythromycin (10000U mLmL-1), to conditionate the media by the prepared materials.

Pulp fibroblast cells were placed in a 96-well plate (20000 cells/well) in triplicate for each specimen, in a total of 9 wells per group. Fibroblasts were kept in DMEM supplemented with 10% FBS and 1% penicillin/erythromycin (10000U mLmL-1), and after 24 h, the cells received the material conditioned media and were incubated for another 24 h. Fibroblasts in fresh medium without NPs extracts were used as a positive control of cell viability, and cells cultured in DMEM with 20% of methanol were considered negative control to cell viability.

Conditioned media were then removed, cells were washed with PBS and incubated for 3 h in DMEM with a 20% solution of MTT at 5 mg/mL in PBS. Media were removed and the insoluble formed crystals were dissolved in 200 µL dimethyl sulfoxide (DMSO) and analyzed at a 570 nm absorbance in a spectrophotometer. 

-*S. mutans* Biofilm Growth on Acquired Pellicle 

The disk-shaped specimens (7 mm diameter and 2 mm in thickness) from each experimental and control group were prepared in triplicate. The disks were kept perpendicularly from the bottom of the wells throughout the whole experiment, with the aid of a metallic device, on 24-well cell culture plates. Acquired pellicle was formed on the surface of the disks after they were immersed in human saliva for 1 h. The saliva that was donated from volunteers (CAAE nº 02893218.2.0000.8084) was previously centrifuged (4000 RPM), diluted in PBS (Laborclin LTDA) at 10% (1:1 in volume) and filtered in 0.22 µm membrane.

For the *Streptococcus mutans* (ATCC 700610) biofilm formation, bacterial starter (1 – 2 x 108 CFU/mL) was suspended in BHI broth (1:1000) with sucrose at 1%. The specimens were first washed in PBS at 10% and then incubated in the *S. mutans* suspension at 37ºC and CO2 5% for 24 h. Disks were incubated for another 48 h in BHI broth and sucrose, having the culture media changed every 24 h. After the 72h biofilm growth, disks were washed in PBS and placed in conical tubes with 3 mL of PBS to be agitated in vortex and in an ultrasonic tank for 10 min. From the contaminated PBS, a serial dilution was performed (10-1–10-6) to enable CFU count. 20 µL of *S. mutans* diluted suspensions (10-4, 10-5 and 10-6) were two times plated in blood agar plates and incubated at 37ºC and CO2 5% for 48 h.

-*S. mutans* Biofilm behavior evaluated by Confocal Laser Scanning Microscopy

Extracellular polysaccharides (EPS) were labeled via incorporation of Alexa Fluor 647 dextran conjugate (D22914, Life Technologies) (absorbance/fluorescence emission maxima of 647/668 nm), while bacterial cells were stained with SYTO 9 (485/498 nm) (S34854, Life Technologies) 30 minutes before confocal imaging. The analysis of intact biofilms was performed using a confocal laser scanning microscope (CLSM - Zeiss LSM 780-NLO, Carl Zeiss Microscopy GmbH) equipped with an EC Plan-Neofluar 63x oil immersion objective lens (excitation wavelength 810 nm). Each biofilm was scanned at least at 5 randomly selected points and a confocal image series was generated by optical sectioning (2 µm intervals) at each of the positions. The confocal image stacks were then analyzed with LAS AF (Leica) software to quantify and characterize the 3D structure of the biofilms.

-Statistical analysis

The data were subjected to normality (Anderson-Darling test) and homoscedasticity analysis (Levine test), and then subjected to one-way ANOVA (CFU, degree of conversion, transmittance values) followed by Tukey’s pairwise comparisons test or Kruskal-Wallis and post-hoc Dunn test (ion release, cytotoxicity assay and delta E analysis). For all cases, the global significance level was 5%.

## Results

-Degree of conversion 

The incorporation of different percentages of Ag@SiO2 NPs tested in this study did not change the degree of conversion of the experimental materials in relation to the control, with data ranging from 83.7 to 85.2% (*p* = 0.566) as shown in Table 1. 

**Table 1:**
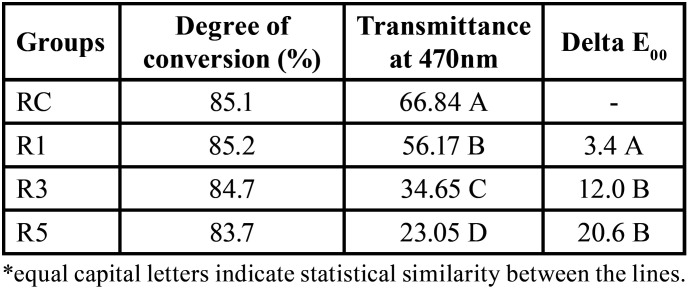
Mean of degree of conversion, transmittance values at 470 nm and median of delta E00, with statistical analysis of the resin materials. RC, Resin control. R1, Resin with 1% of Ag@SiO2 NPs. R3, Resin with 3% of Ag@SiO2 NPs. R5, Resin with 5% of Ag@SiO2 NPs.

-Optical properties 

Significant reductions were observed in light transmittance with the incorporation of Ag@SiO2 NPs (*p*<0.01). Transmittance values at 470 nm (Table 1) decreased by up to one third (R5 group = 23%) compared to the control (RC group = 67%).

Like what was observed for the transmittance data, the incorporation of Ag@SiO2 NPs significantly changed (*p*<0.01) the ∆E00 values (Table 1). The incorporation of 1% Ag@SiO2 NPs provided significantly smaller color changes than the other percentages evaluated. The groups presented values of ∆E00 ranging between 3.4 e 20.6 (R1and R5 groups, respectively).

-Microstructural analysis

**Figure 4:**
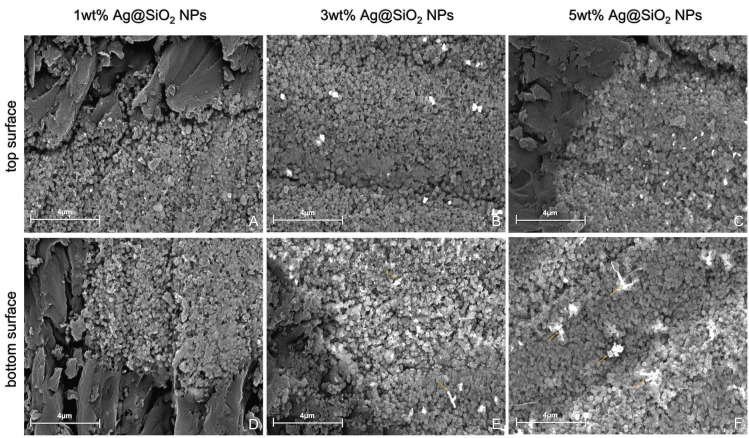
FEG-SEM images from top and bottom of the experimental groups of materials containing different percentages of Ag@SiO2 NPs. Silver nanoparticles can be visualized as white points in all images and arrows point to particle aggregates. A,D, Top and then bottom surface of disk with 1wt% Ag@SiO2 NPs. B,E, Top and then bottom surface of disk with 3wt% Ag@SiO2 NPs. C,F, Top and then bottom surface of disk with 5wt% AgSiO2 NPs.

Figure 4 shows the images (top and bottom surface) obtained at 30,000 times magnification for all materials containing Ag@SiO2 NPs. Silver nanoparticles can be easily visualized in the images, due to their contrast difference with the other components of the materials. Regular spherical silica particles are also observed with an average diameter of 0.26 (+ 0.07) µm. In Figure 4E, F it is possible to observe silver aggregates shaped like a rod with 0.89 (+ 0.28) µm of length.

-Silver ion release

Ion release data are presented in Table 2. R3 and R5 showed statistically similar results at 24h, higher than R1 (*p*<0.01). Silver release decreased over time and at 72 h concentrations were below ICP-OES detection limit.

**Table 2:**
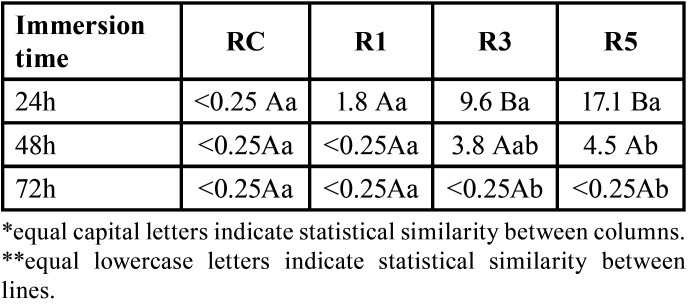
Median and statistical analysis of silver ion release values in parts per billion (ppb, µgL-1) of the resin materials. RC, Resin control. R1, Resin with 1% of Ag@SiO2 NPs. R3, Resin with 3% of Ag@SiO2 NPs. R5, Resin with 5% of Ag@SiO2 NPs.

-Cytotoxicity assay

All groups containing Ag@SiO2 NPs revealed a slide cell viability effect. No statistically significant differences were found among the groups treated with the 1, 3 and 5 wt% of Ag@SiO2 NPs, nor with the controls (RC or no treatment).

-Ag@SiO2 NPs performance on *S. mutans* biofilm 

**Figure 5:**
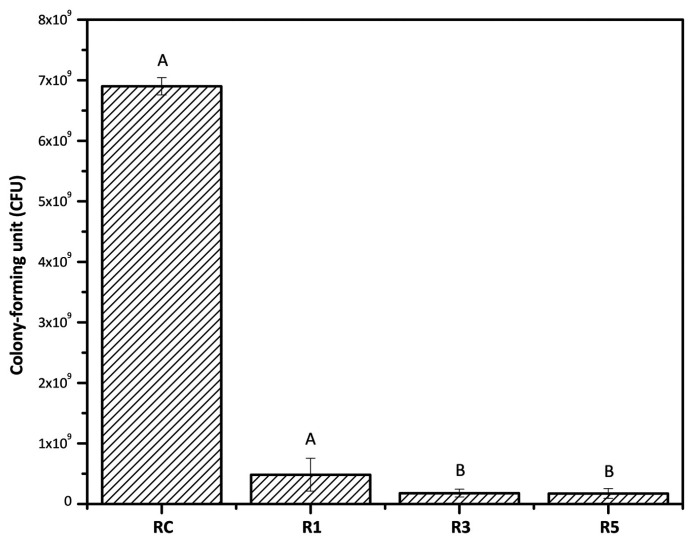
*S. mutans* colony-forming units (CFU) count results after 72h of growth, for all materials. RC, Resin control. R1, Resin with 1% of Ag@SiO2 NPs. R3, Resin with 3% of Ag@SiO2 NPs. R5, Resin with 5% of Ag@SiO2 NPs) with statistical analysis (equal capital letters indicate statistical similarity between groups).

The addition of Ag@SiO2 NPs in the experimental resin reduced *S. mutans* biofilm formation on acquired pellicles for all evaluated groups, as presented in Figure 5. After 72 h, the CFU count on the surface of the R1 resin samples decreased to 4.8x108 CFU in comparison to the RC group (6.9x109 CFU), without statistical difference. With RC3 and RC5 showed a similar behavior, with statistically significant reductions in colony formation of 1.8 x 108 and 1.7 x 108, respectively (*p*<0.05).

-Biofilm evaluation by Confocal Laser Scanning Microscopy 

**Figure 6:**
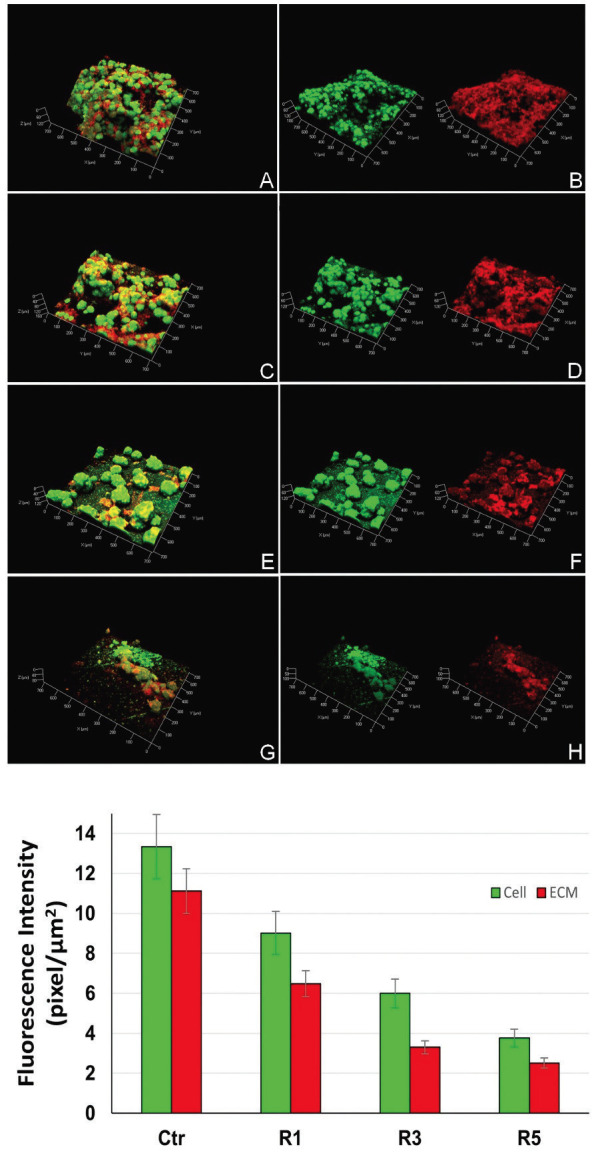
Confocal Laser Scanning Microscopy: Bacterial cells (green) and Extracellular polysaccharides (EPS) (red) behavior was evaluated before and after treatment with the materials. A, B, Merged image and then cells and EPS images from control resin. C, D, Merged image and then cells and EPS images from resin with 1% of Ag@SiO2 NPs. E, F, Merged image and then cells and EPS images from resin with 3% of Ag@SiO2 NPs. G, H, Merged image and then cells and EPS images from resin with 5% of Ag@SiO2 NPs. The graph corresponds to the emitted fluorescence intensity by the bacterial cells colonies (green channel) and the extracellular polysaccharides (red channel) quantified as pixel/µm.

The bacterial cells and EPS showed a considerable decrease in the number of colonies and biofilm thickness in all treated samples in Figure 6. Biofilms exposed to 1% (Fig. 6C,D), 3% (Fig. 6E,F), and 5wt% (Fig. 6G,H) of Ag@SiO2 NPs were able to promote reduction in the EPS layer as well as in the colonies number and structure. The fluorescence intensity quantification (Fig. 6) showed that the Ag@SiO2 NPs act in the biofilm formation in a dose-dependent way both in bacterial cells and in EPS.

## Discussion

Biochemical and molecular techniques have demonstrated the mechanism of antimicrobial activity of AgNPs (30,31). The antimicrobial activity of AgNPs is related to four well-defined mechanisms: i) adhesion of AgNPs onto the surface of bacterial wall and membrane, ii) AgNPs penetration and damaging of intracellular structures, such as lysosomes, mitochondria, vacuoles, ribosomes, and biomolecules (protein, lipids, and DNA), iii) induction of cellular toxicity by generation of reactive oxygen species (ROS) and free radicals consequence of oxidative stress, and iv) modulation of signal transduction pathways. Moreover, AgNPs modulate the immune system of the human cells by orchestrating inflammatory responses, which further aid in inhibition of microorganisms (32).

In this study, it was observed that the higher the percentage of Ag@SiO2 NPs, the lower the transmittance value and, consequently, the light passage. However, even with lower transmittance values in the experimental groups (56, 35 and 23% for the concentrations of 1, 3 and 5% of Ag@SiO2 NPs, respectively), the degree of conversion of resin materials was like that of the group control.

Nevertheless, the coating of AgNPs with silica was not able to effectively mask the color change that the incorporation of these nanoparticles causes in the resin material. The ∆E_00 data of the groups containing Ag@SiO2 NPs varied between 3.4 and 20.6, exceeding the color change perceptibility limit for the methodology used (CIEDE 2000) which is 1.8 (29). Most studies that performed the incorporation of silver nanoparticles in resin materials do not objectively evaluate the color change that the material presents (1,2,18,20). However, it is worth noting that the existing literature includes reports on the assessment of color alteration in dental substrates following the utilization of silver-containing resins (33). These aspects are crucial for understanding the practical implications of these materials in clinical applications. The ∆Ε (CIELab formula) values ranging between 18.5 and 32.1 were observed for concentrations of 0.24 and 0.36% by mass of AgNPs (26), corresponding to those observed in the present study for the groups containing 3 and 5% in mass of Ag@SiO2 NPs (∆Ε = 18 and 28, respectively). The particles exhibited distribution within the resinous material, with a noTable accumulation at the base of the specimens. This phenomenon can be attributed to their deposition, resulting from their higher weight compared to the resin matrix.

The analysis of silver ion release was conducted to estimate its relationship with the antimicrobial activity and/or possible cytotoxic effect of the materials. The increased ion release did not lead to an increase in antimicrobial activity at the highest concentrations of Ag@SiO2 NPs studied (3 and 5wt%), suggesting the plateau effect already discussed. Regarding the cytotoxicity of the materials, it was observed that the incorporation of Ag@SiO2 NPs did not significantly change the cytotoxicity of the materials in relation to the control material. However, all materials containing Ag@SiO2 NPs showed cell viability below 70%, demonstrating a tendency towards cytotoxicity. A possible justification for these findings is that these polymeric materials, with small percentages of inorganic particles (in this case, only Ag@SiO2 NPs), can absorb more water (34), and thus can facilitate the transfer of silver ions from the material to the medium, increasing cytotoxicity (35).

Data from the CFU analyses showed that Ag@SiO2 NPs content of 3 and 5wt% was able to significantly reduce bacterial growth as well as shown in the confocal microscopy evaluation, in a dose-dependent pattern. Contrary to what might be expected, the increase in the number of silver particles did not result in greater antibacterial activity. This plateau effect observed with increasing concentrations from 3 to 5 wt% of AgNPs is not uncommon (20,26) and can be attributed to the fact that, in higher concentrations, nanoparticles tend to agglomerate, reducing surface availability that would favor antimicrobial activity (20).

Percentages from 3 wt% of Ag@SiO2 NPs provided significant reductions (approximately 75%) in the CFU count of *S. mutans* in oral biofilm, on the resin material. The literature reports even more expressive results, such as a 94% reduction in active colonies of this same pathogen, when 1wt% of silver nanoparticles were incorporated (9). However, it is necessary to emphasize that in the present work antimicrobial activity was evaluated after the formation of an acquired pellicle as well the inclusion of sucrose in the medium, exposing the materials to a much more complex condition.

Within this perspective, some factors must still be considered: i) the antimicrobial analysis performed involves biofilm formation in the presence of fermentable sugar, which guarantees the formation of a matrix rich in EPS that hinders the arrival of the antimicrobial agent to the pathogen (24); ii) the coating of AgNPs with silica implies a lower effective content of silver particles since silica represents about 18% of the total mass of Ag@SiO2 NPs (27), thus the amount of silver effectively available in the experimental groups was 0.82, 2.46 and 4.1 wt% in groups 1, 3 and 5% respectively; iii) finally, the silica coating may have reduced the direct contact of microorganisms with the antimicrobial agent, which could minimize its effectiveness since one of the mechanisms of action of silver suggested in the literature is through contact inhibition (22).

Thus, it is estimated that there are two strategies to be explored in future work to achieve better results: reduction of the percentage of Ag@SiO2 NPs incorporated in the material and subsequent evaluation of the modulation of biofilm growth with *S. mutans*; and/or modification of the composition of the resin material, through the incorporation of inorganic particles (barium glass and/or silica), reducing the resin matrix volume, and consequently the material’s water sorption.

The use of metallic nanoparticles coated with silica is still little explored in the development of dental materials. The data obtained so far demonstrate an important potential for the application of Ag@SiO2 NPs, yet with adjustments to be made. More studies should be conducted using lower percentages of Ag@SiO2 NPs aiming to minimize the adverse effects of cytotoxicity and color change of materials, seeking the modulation of oral biofilm, as already pointed out in recent works(36). Furthermore, the modification of the composition of the resin material, through the incorporation of inorganic particles, also deserves to be explored, benefiting from the characteristics they bring to the material.

## Conclusions

In summary, the study shows that the application of 3 wt% Ag@SiO2 NPs in a resinous matrix demonstrates a significant reduction of *S. mutans* biofilm, The incorporation of Ag@SiO2 NPs did not increase the cytotoxicity of the resinous materials, however all materials with NPs showed a tendency towards cytotoxicity in dental pulp fibroblasts. The transmittance values of the resins containing Ag@SiO2 NPs were significantly lower than the control, but without affecting their degree of conversion. Additionally, all percentages of Ag@SiO2 NPs incorporated represented significant color change in the materials, being one of the challenges still to be overcome.
